# Intestinal mRNA expression analysis of polarity‐related genes identified the discriminatory ability of CRB3 as a diagnostic marker for celiac disease

**DOI:** 10.1002/iid3.1186

**Published:** 2024-02-14

**Authors:** Tannaz Taraz, Nastaran Asri, Ehsan Nazemalhosseini‐Mojarad, Flora Forouzesh, Mostafa Rezaei‐Tavirani, Mohammad Rostami‐Nejad

**Affiliations:** ^1^ Department of Genetics, Faculty of Advanced Science and Technology, Tehran Medical Sciences Islamic Azad University Tehran Iran; ^2^ Gastroenterology and Liver Diseases Research Center, Research Institute for Gastroenterology and Liver Diseases Shahid Beheshti University of Medical Sciences Tehran Iran; ^3^ Basic and Molecular Epidemiology of Gastrointestinal Disorders Research Center, Research Institute for Gastroenterology and Liver Diseases Shahid Beheshti University of Medical Sciences Tehran Iran; ^4^ Proteomics Research Center, Faculty of Paramedical Sciences Shahid Beheshti University of Medical Sciences Tehran Iran; ^5^ Celiac Disease and Gluten Related Disorders Research Center, Research Institute for Gastroenterology and Liver Diseases Shahid Beheshti University of Medical Sciences Tehran Iran

**Keywords:** celiac disease, gluten, gluten free diet, inflammation, polarity complexes

## Abstract

**Background:**

Celiac disease (CD) is a chronic autoimmune disorder characterized by an abnormal immune response to gluten, a protein found in wheat, barley, and rye. It is well established that the integrity of epithelial tight junctions (TJs) and adherens junctions (AJs) plays a crucial role in the pathogenesis of CD. These junctional complexes contribute to the apical–basal polarity of the intestinal epithelial cells, which is crucial for their proper functioning.

**Methods:**

Sixty CD subjects, and 50 controls were enrolled in the current study. Mucosal samples were obtained from the distal duodenum, total RNA was extracted and complementary DNA was synthesized. The relative expression levels of the desired genes were evaluated by quantitative real‐time polymerase chain reaction based on ΔΔ*C*_*t*_ method. The gene–gene interaction network was also constructed using GeneMANIA.

**Results:**

*CRB3* (*p* = .0005), *LKB1* (*p* < .0001), and *SCRIB* (*p* = .0005) had lower expression in CD patients compared to controls, while *PRKCZ* expression did not differ between groups (*p* > .05). *CRB3* represented a significant diagnostic value for differentiating CD patients from the control group (*p* = .02).

**Conclusion:**

The aim of the current study was to evaluate the changes in the mRNA expression levels of *SCRIB*, *PRKCZ*, *LKB1*, and *CRB3* genes in the small intestinal biopsy samples of CD patients in comparison to the healthy control subjects. Our data uncover the importance of polarity‐related genes (especially *CRB3*) in CD pahtomechanism, that may facilitate the planning of the future studies looking for finding innovative diagnostic and therapeutic strategies for CD.

## Graphical abstract

Intestinal epithelial apical–basal polarity plays a pivotal role in controlling epithelial AJs and TJs. Our data uncover the importance of polarity‐related genes (especially PRKCZ) in CD pahtomechanism, that may facilitate the planning of the future studies looking for finding innovative diagnostic and therapeutic strategies for CD.

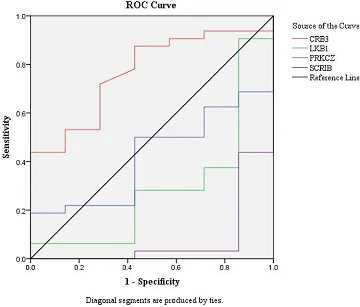

## 1 INTRODUCTION

Celiac disease (CD), is an autoimmune intestinal disorder mediated by aberrant immune responses to dietary gluten proteins.^1^, ^2^ The pathogenesis of CD involves inflammation and immune dysregulation, which contribute significantly to the development of the disease. A key factor in CD pathogenesis is the loss of integrity of the intestinal epithelial barrier, which is a result of inflammation.^3^, ^4^ Indeed, the impairment of the intestinal epithelial barrier leads to alterations in the positioning of both epithelial tight junctions (TJs) and adherens junctions (AJs). Consequently, there is an increased entrance of semi‐digested gluten peptides to the lamina propria, leading to the inflammatory responses.^5^ CD may, therefore, present with gastrointestinal symptoms related to inflammation and nutrient deficiency due to damage to the small intestinal mucosa and villous atrophy.^6^ The polarity of the intestinal epithelial apical–basal axis plays a pivotal role in maintaining epithelial barrier structure, which controls epithelial AJs and TJs.^7^, ^8^ The establishment and maintenance of apical‐basolateral polarity are governed by three prominent and evolutionarily conserved complexes known as the Scribble cell polarity complex, the Crumbs cell polarity complex, and the Par cell polarity complex.^9^ The Scribble cell polarity complex consists of SCRIB, DLG, and LGL proteins and is located in the basolateral membrane.^10^ SCRIB is a conserved polarity protein with an important role in maintaining epithelial polarity.^10^, ^11^ SCRIB, an essential scaffolding protein involved in critical cellular processes such as epithelial cell differentiation and tumor suppression, exhibits prominent expression in the intestinal tissue.^12^ The Crumbs cell polarity complex consists of CRB3, PALS1, and PATJ. CRB3 interacts with PALS1 and PATJ, forming a three‐component complex that plays a role in the formation and stability of TJs.^13^ The Par cell polarity complex consists of Par‐3 and Par‐6, atypical protein kinase C (aPKC) including PKCζ (PRKCZ) and PKCλ, and a small protein with GTPase property called CdC42.^14^ PKCζ in conjunction with other Ser/Thr kinases, possesses the capability to phosphorylate occludin at distinctive residues. The Ser/Thr‐phosphorylated occludin interacts with other TJ proteins and maintain intestinal barrier integrity.^15^, ^16^ Par4 (LKB1) is also a Ser/Thr kinase whose activation is sufficient to induce polarity in intestinal epithelial cells, even in the absence of apical junctions.^17^, ^18^ Missense mutations leading to the loss of Par‐4 function have been identified in different types of cancers, such as colon cancer and small intestine cancer, which have been reported to be linked to CD.^19^, ^20^

Actually, the apical–basal axis of polarity is known as a central organizer of intestinal epithelial barrier integrity that forms impermeable cell–cell junctions, and its defects can have profound effects on tissue homeostasis, including inflammation and immune responses and contribute to the pathogenesis of CD.^17^, ^20^

Strict adherence to a gluten‐free diet is currently the only treatment option for individuals with CD, which can be challenging for patients and may potentially have negative impacts on their physical characteristics. Consequently, researchers are exploring various aspects of the disorder's pathogenesis to discover new supplementary therapeutic approaches for CD patients.^21^, ^22^, ^23^ Since the disruption of apical–basal polarity and the integrity of the barrier are crucial factors in the pathogenesis of CD, exploring alterations in the expression of genes related to the apical–basal polarity complex in CD patients could offer valuable insights for future research. This approach may aid in identifying novel therapeutic targets for CD. In our study, we analyzed the variations in mRNA expression of *SCRIB*, *CRB3*, *LKB1*, and *PRKCZ* genes in CD patients compared to healthy individuals serving as control subjects.

## 2 MATERIAL AND METHODS

### 2.1 Study design and sample collection

We included 60 CD patients (50 treated and 10 active CD) who underwent serological tests and duodenal biopsy evaluations, and were referred to the Celiac Disease and Gluten‐Related Disorders Research Center, Shahid Beheshti University of Medical Sciences between 2021 and 2023. Moreover, 50 healthy individuals without any history of CD and other autoimmune diseases up to their first‐degree relatives, who underwent the endoscopy because of other gastrointestinal symptoms, but had normal small intestinal biopsy specimens included as a control group. Subjects with any type of chronic diseases of the upper gastrointestinal tract, who suffered from cancers or *Helicobacter pylori* infection, and pregnant/lactating women were excluded from the present study. Duodenal biopsies of small intestine were collected from these three groups and kept in RNA later at a temperature of −70°C for further evaluation.

### 2.2 RNA extraction and complementary DNA synthesis

In this study, YTA Total RNA Purification Mini kit for Blood/Cultured Cell/Tissue kit (Yekta Tajhiz Azma) was used to extract the total RNA from small intestinal biopsy specimens. The purity and concentration of the extracted RNA was determined by the NanoDrop‐1000 spectrophotometer (NanoDrop Technologies). The 2 Step 2X RT‐PCR Premix (Taq) kit (BioFact™) was used to reverse transcription of RNA into complementary DNA (cDNA).

### 2.3 Primer designing and reverse‐transcription quantitative real‐time polymerase chain reaction (RT‐qPCR)

Specialized software such as Gene runner and Primer3 were used to design specific primers for *SCRIB*, *CRB3*, *LKB1*, *PRKCZ*, and Beta2 micro‐globulin (*β2M*), as house‐keeping gene. Well‐designed primers were synthesized by Pishgam corporation. The characteristics of designed primers are shown in Table S1.

The mRNA expression levels of *SCRIB*, *CRB3*, *LKB1*, *PRKCZ*, and *β2M* were quantified by real‐time PCR. Real‐time qPCR reactions were carried out in a final volume of 20 μL using SYBR Premix Ex Taq (RealQ Plus 2x Master Mix Green‐Amplicon). The Rotor‐Gene Q series was used and reactions were performed under the following conditions: Initial denaturation 15 min at 95°C, followed by 40 cycles of 20 s at 95°C and 60 s at 60°C (for *SCRIB*, *CRB3*, and *LKB1*) and at 59°C (for *PRKCZ*). The $2^{-∆∆C_{t}}$ method was used to determine changes in gene expression levels $\left(∆C_{t}=C_{t_{\mathrm{target}}}-C_{t_{\mathrm{house}\;\;\;\mathrm{keeping}}}\right)$.

### 2.4 Analysis of gene–gene interactions (GGIs) using GeneMANIA database

We used GeneMania (http://www.genemania.org/) for generating a GGI network to find *SCRIB*, *CRB3*, *LKB1*, and *PRKCZ* related genes, which allowed us to make novel predictions about their important targets that might be important in CD pathogenesis.^24^

### 2.5 Statistical analysis

Data were analyzed using IBM SPSS Version 21 (SPSS Inc.) and PRISM software version 5.00. Mean ± standard deviation (SD) was used for expressing the data. Student *t* test was used to analyze the differences between the means of studied groups. The correlation coefficient between variables was evaluated by using the Spearman and Pearson correlation tests. The receiver operating characteristic (ROC) curve was also used to assess the *SCRIB*, *CRB3*, *LKB1*, and *PRKCZ* PB mRNA expression diagnostic value for distinguishing CD patients from healthy controls. A *p* value less than .05 is deemed to be statistically significant.

## 3 RESULT

### 3.1 Demographic and clinical characteristics of study groups

Table 1 presents the characteristics of both the CD patients and the control group. Despite there being no significant difference in age and sex between the patients and controls (*p* > .05), there was a significant difference in the median BMI between the two groups (*p* = .03).

**Table 1:** Demographic characteristics of study participants.

Variables		Groups			
		Controls	CD patients		*p* Value
Number		50	60		
Gender					
Male		21 (42%)	24 (40%)		.36
Female		29 (58%)	36 (60%)		
Age		39.02 ± 11.24	38.42 ± 14.05		.8
BMI		27.3 ± 3.42	22.67 ± 5.28		.03*
Intestinal damage (Marsh classification)			Marsh I	3 (5%)	
			Marsh II	26(43.3%)	
			Marsh III	31 (51.6%)	

Abbreviations: BMI, body mass index; CD, celiac disease.

* Statistically significant.

**Figure 1:**
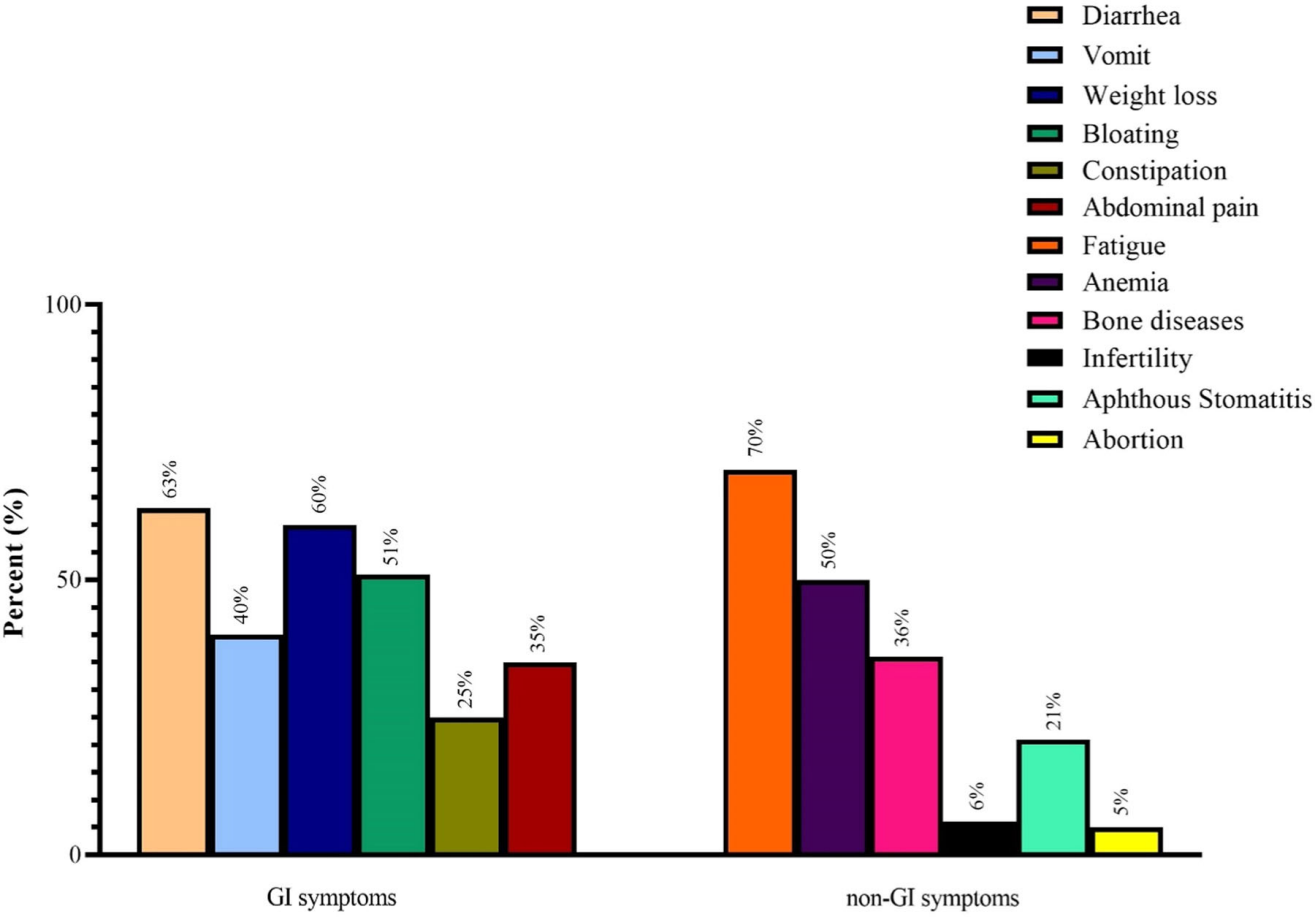
Gastrointestinal (GI) and non‐GI symptoms reported by celiac disease (CD) patients: most patients complained of two or more symptoms.

The most common gastrointestinal symptom of CD patients were diarrhea (63%). Furthermore, the most common non‐gastrointestinal symptom of them was fatigue (70%) (Figure 1).

### 3.2 mRNA expression of *SCRIB*, *CRB3*, *LKB1*, and *PRKCZ*

**Figure 2:**
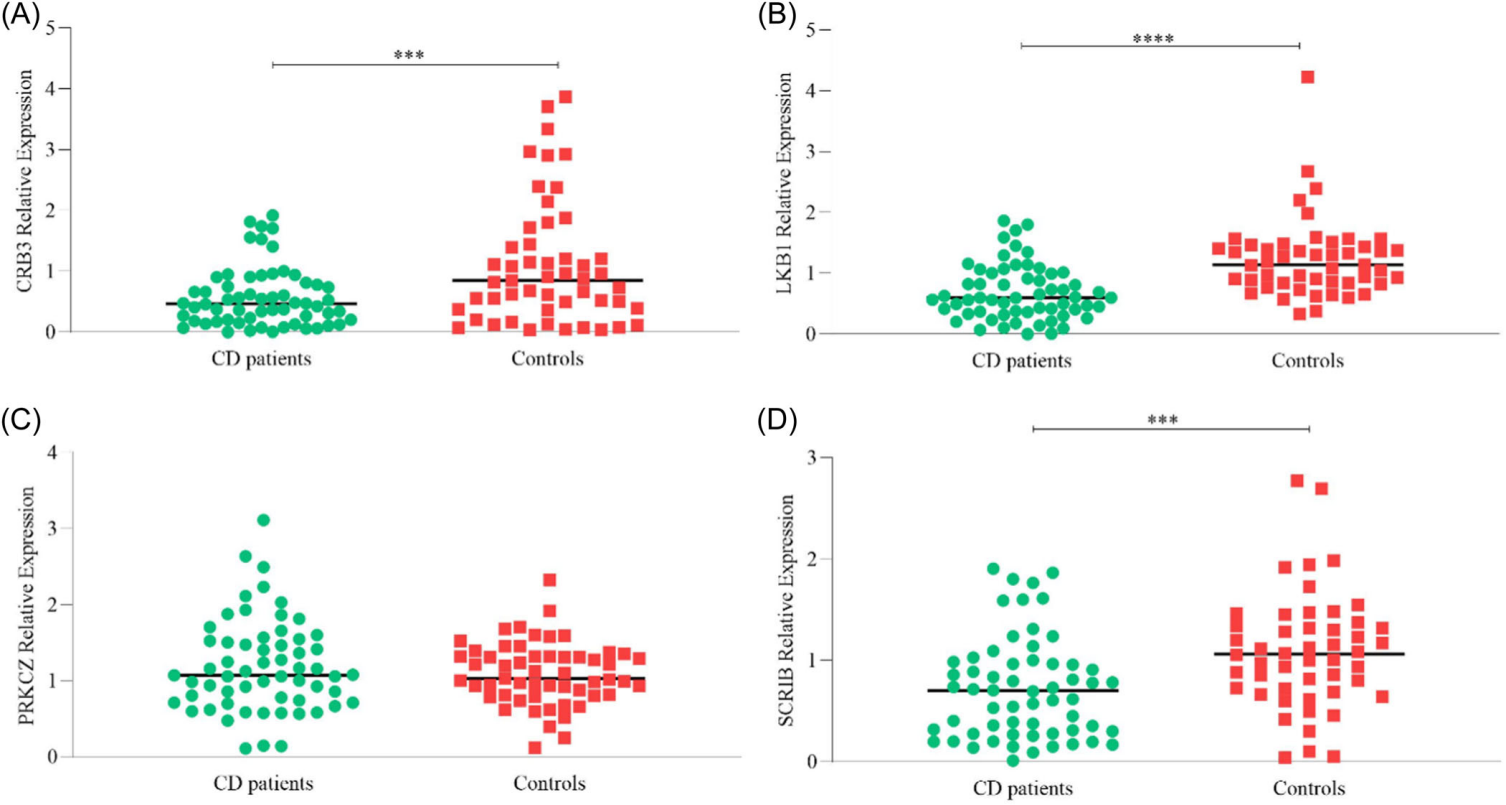
Analysis of relative expression levels in celiac disease (CD) patients (*n* = 60) compared to controls (*n* = 50) using real‐time polymerase chain reaction (PCR) assay. All expression levels are normalized to that of *β2M*. The analyzed genes were as follows: (A) *CRB3*; ***p = .0005, (B) *LKB1*; ****p < .0001, (C) *PRKCZ*, (D) *SCRIB*; ***p = .0005.

We investigated the mRNA expression of *SCRIB*, *CRB3*, *LKB1*, and *PRKCZ* in duodenal biopsy specimens of CD patients and controls using *β2M* as housekeeping gene. According to our results, *CRB3* (*p* = .0005), *LKB1* (*p* < .0001) and *SCRIB* (*p* = .0005) had significantly lower expression in CD patients than in controls. *PRKCZ* mRNA expressions did not differ between cases and controls (*p* > .05) (Figure 2).

### 3.3 Correlation analysis

**Figure 3:**
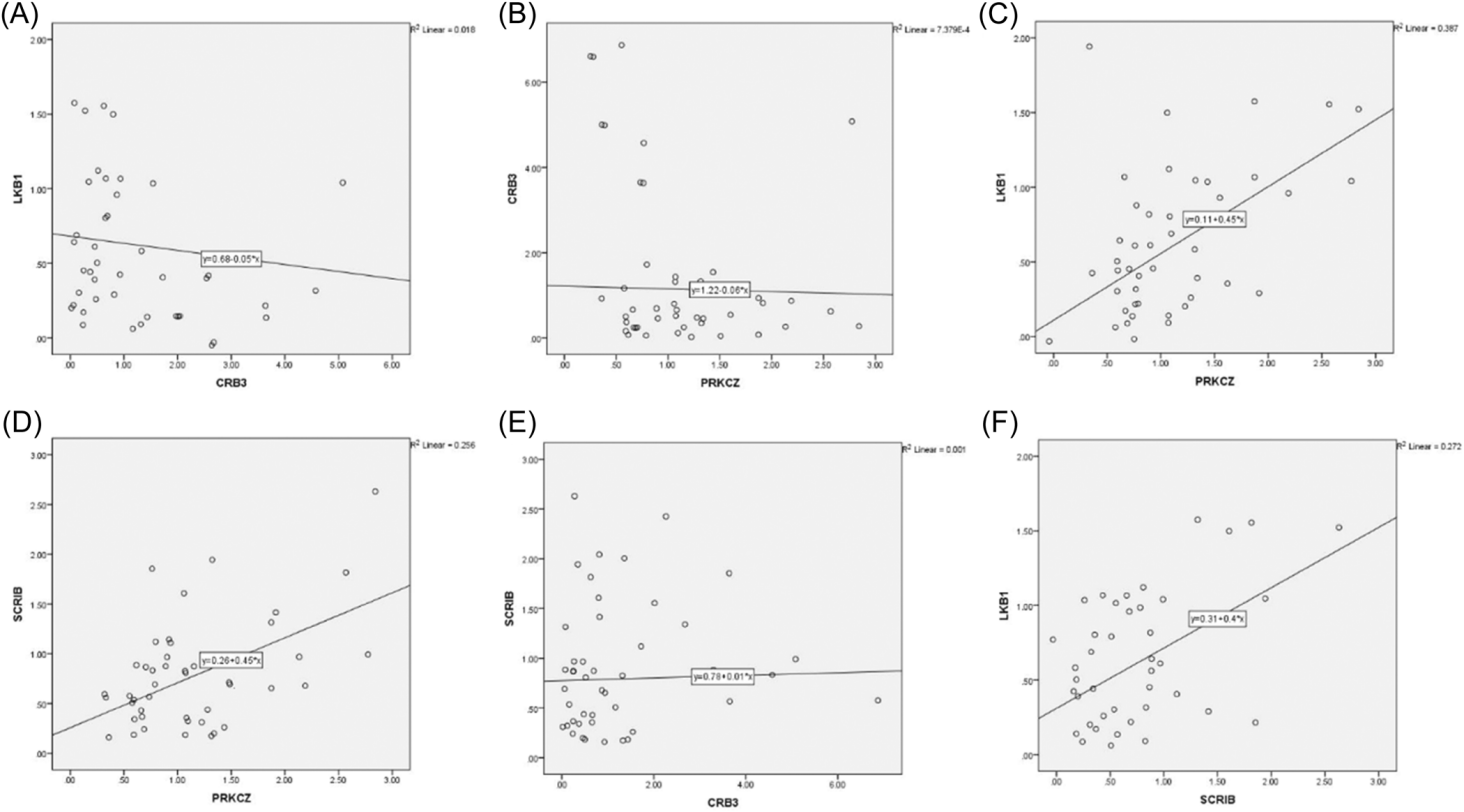
Scatter plots representing the correlation between cell polarity‐related genes mRNA expression in celiac disease (CD) patients (*n* = 60). (A) Pearson correlation analysis between LKB1 and CRB3. (B) Correlation between PRKCZ and CRB3. (C) Correlation between PRKCZ and LKB1. (D) Correlation between PRKCZ and SCRIB. (E) Correlation between SCRIB and CRB3. (F) Correlation between CRB3 and LKB1. The regression line is also represented (Pearson correlation coefficient [*r*], *p* value [*p*]).

Spearman and Pearson correlation tests were used to evaluate the correlation between *SCRIB*, *CRB3*, *LKB1*, and *PRKCZ* mRNA expressions and the relationship between clinical symptoms and intestinal tissue expression levels of those genes in CD patients. As shown in Figure 3, the relative expression of *LKB1* was positively correlated with that of *PRKCZ* (*r* = .62, *p* = .01, Figure 3C) and *SCRIB* (*r* = .52, *p* = .001, Figure 3F). Moreover, there was a significantly positive correlation between *PRKCZ* and *SCRIB* mRNA expressions (*r* = .5, *p* = .001, Figure 3D). The correlation between other genes was not statistically meaningful.

**Figure 4:**
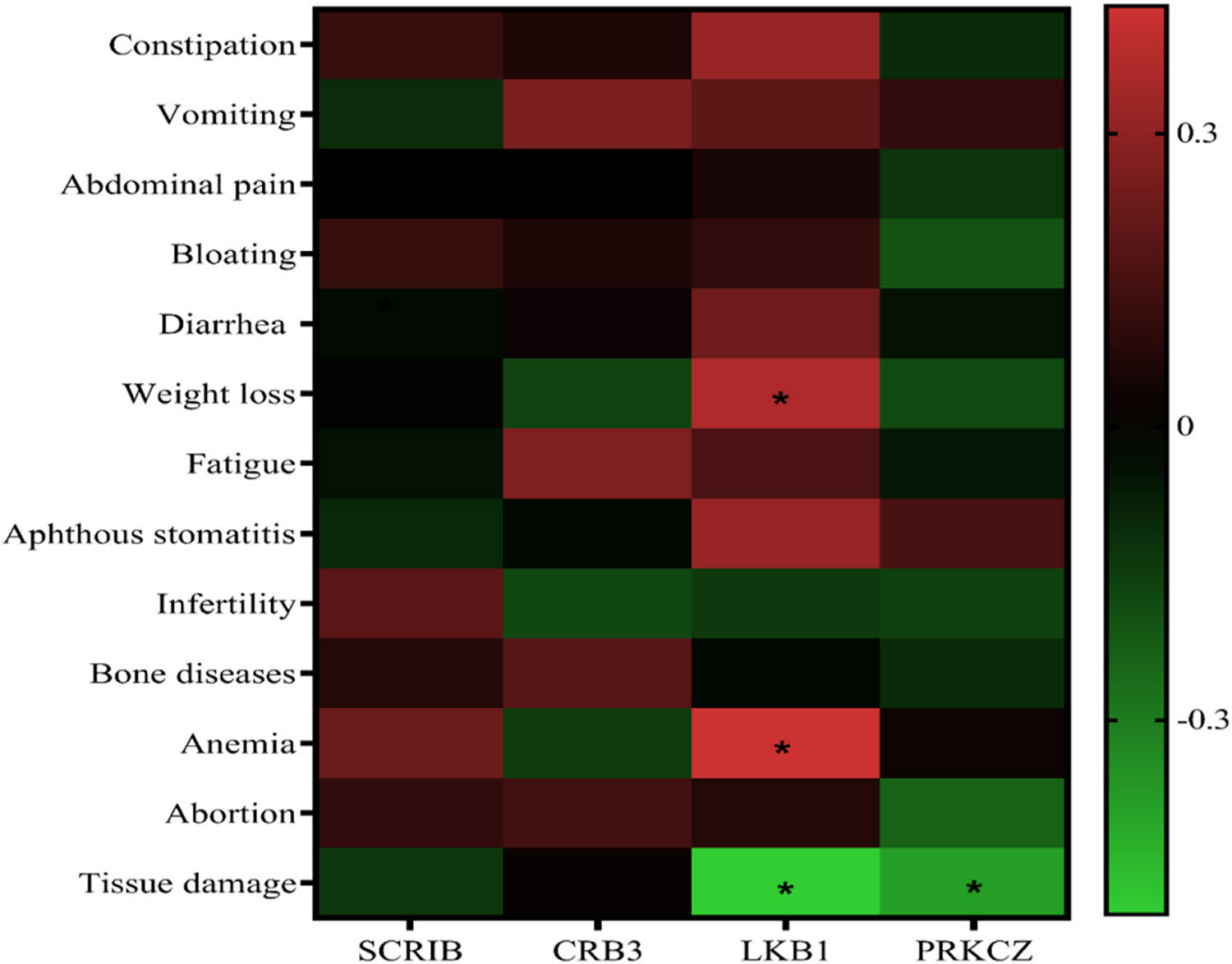
A heat map of Pearson correlation between duodenal mRNA expression levels of *SCRIB*, *CRB3*, *LKB1*, *PRKCZ*, and patients clinical and pathological characteristics. Red: positive correlations; green: negative correlations (heat map presents *r* values).

Spearman correlation test was used to investigate the relationship between clinical symptoms and intestinal tissue expression levels of *LKB1*, *SCRIB*, *PRKCZ*, and *CRB3* genes. According to the findings, there were significant positive correlations between *LKB1* mRNA expression and anemia (*p* = .008, *r* = .430) and weight loss (*p* = .026, *r* = .365). *LKB1* (*p* = .002, *r* = .499), and *PRKCZ* (*p* = .017, *r* = .389) showed significant negative correlations with the severity of the tissue damage (Figure 4).

### 3.4 ROC curve analysis

**Figure 5:**
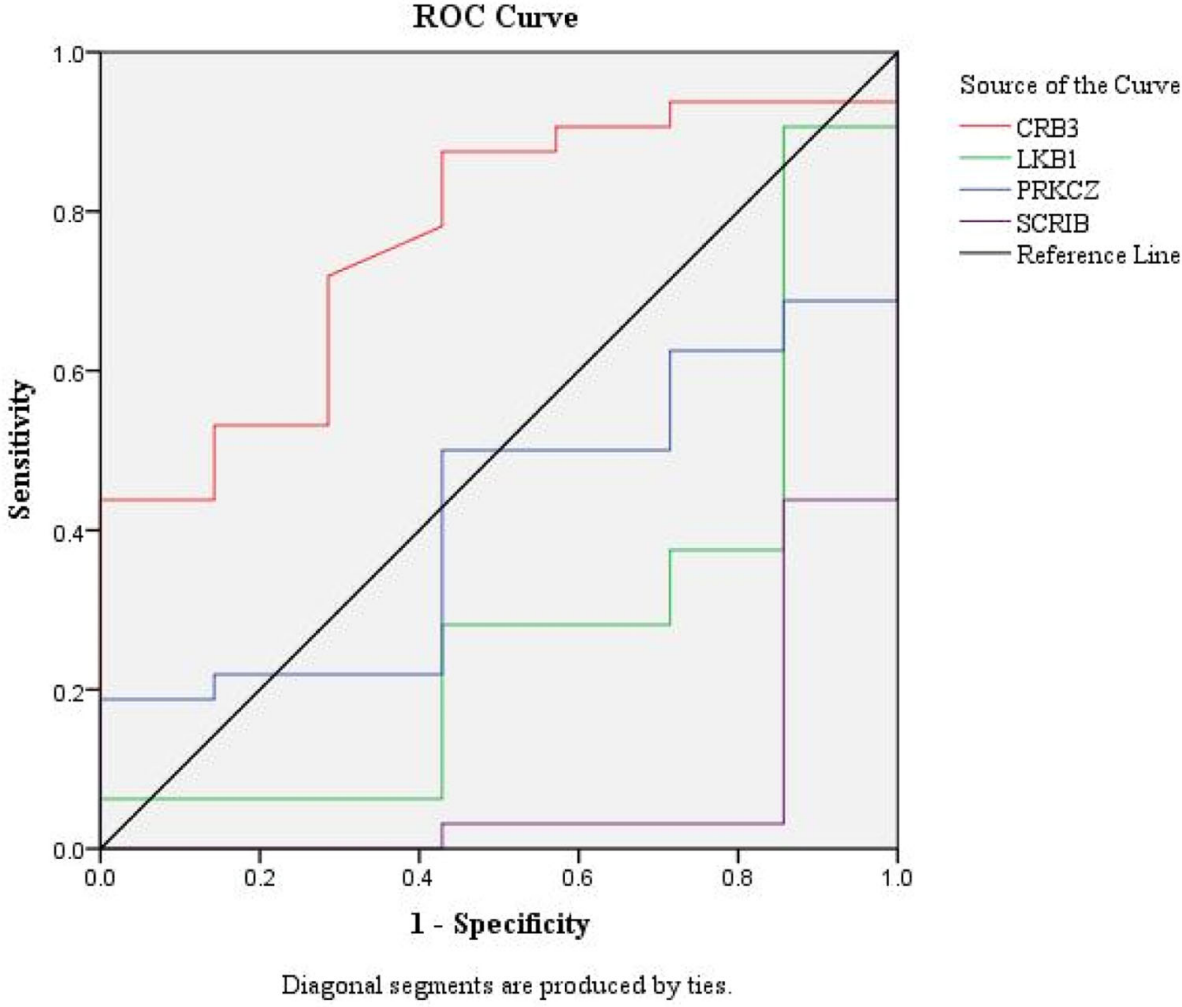
Receiver operating characteristic (ROC) curve analysis of *CRB3*, *LKB1*, *PRKCZ*, and *SCRIB* duodenal mRNA levels for distinguishing between celiac disease (CD) patients and controls.

To assess the diagnostic value of *SCRIB*, *CRB3*, *LKB1*, and *PRKCZ* mRNA expression in distinguishing active CD patients from controls (according to the area under the curve [AUC]), ROC curve analyses were performed. The Youden index was used to select the optimum cutoff values. As shown in Figure 5 and Table 2, the duodenal mRNA level of *CRB3* had the highest AUC (0.76) and represented a significant diagnostic value for CD (*p* = .02).

**Table 2:** Diagnostic performance of the selected studied genes.

	AUC	Sensitivity (%)	Specificity (%)	cutoff value	Youden's *J* index
CRB3	0.76	87	57	0.39	0.44
LKB1	0.29	6	100	1.64	0.06
PRKCZ	0.42	18	100	1.33	0.18
SCRIB	0.07	100	0	3.31	0

Abbreviation: AUC, Area under the curve.

### 3.5 Analysis of gene interaction of CRB3, LKB1, PRKCZ, and SCRIB

**Figure 6:**
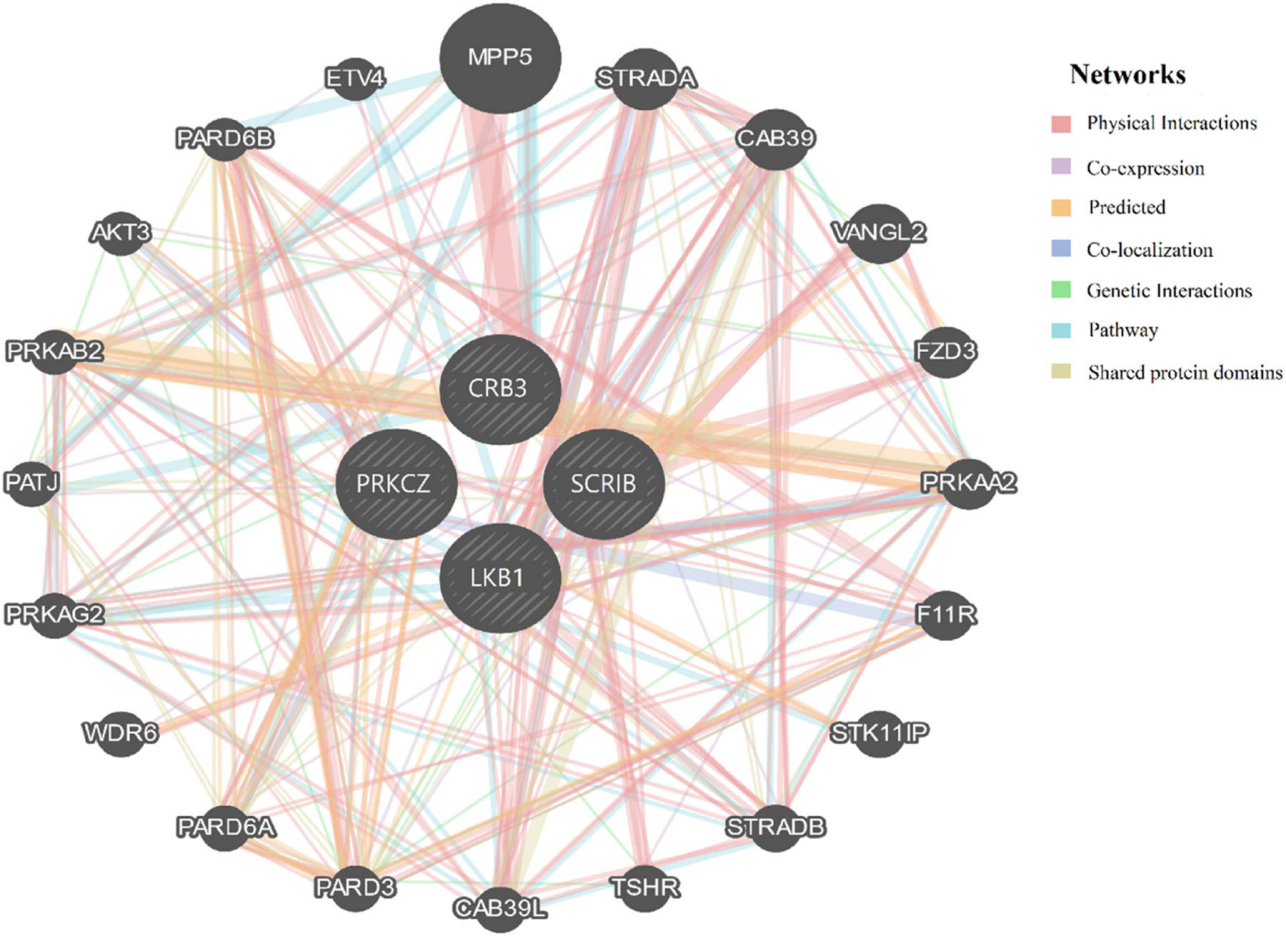
Gene–gene interaction networks constructed by GeneMANIA. Each node in the figure represents a gene, and the size of the node indicates the degree of interaction. Connecting lines between nodes represent gene–gene interactions and their colors represent the type of interaction.

GeneMANIA was used to construct a GGI network composed of *SCRIB*, *CRB3*, *LKB1*, and *PRKCZ*. These four genes were surrounded by 20 nodes, representing genes that may have physical interactions, co‐expressions, predictions, co‐localizations, genetic interactions, pathways, and shared protein domains with them. As shown in Figure 6, the most relevant genes were membrane protein palmitoylated 5 (*MPP5*)/*PALS1*, STE20 related adaptor alpha (*STRADA*), calcium binding protein 39 (*CAB39*), VANGL planar cell polarity protein 2 (*VANGL2*), frizzled class receptor 3 (*FZD3*), protein kinase AMP‐activated catalytic subunit alpha 2 (*PRKAA2*), F11 receptor (*F11R*), serine/threonine kinase 11 interacting protein (*STK11IP*).

## 4 DISCUSSION

According to our results, *SCRIB*, *LKB1*, and *CRB3* had a significant reduction in intestinal mRNA levels of our CD patients in comparison to the controls. To the best of our knowledge, this is the first report regarding the intestinal expression of *SCRIB* and *LKB1* in CD patients, and further studies are needed to confirm these results. In a 2020 study by Breugelmans and colleagues, they explored inflammatory bowel disease (IBD), a type of intestinal inflammation. The researchers discovered that overexpression of membrane mucins had a significant impact on the expression of genes associated with apical‐basolateral polarity, such as *SCRIB*, *CRB3*, and *aPkcζ*. This, in turn, resulted in a dysfunctional epithelial barrier and alterations in the permeability of cell junctions.^25^ Mashukova et al. revealed that the loss of *aPKC* or *Par3* function during intestinal inflammation was accompanied by increased *NF‐κB* activity and *TNF‐α* response, leading to increased paracellular leakage.^26^ Same as we observed, Jauregi‐Miguel and colleagues in 2014, indicated that *CRB3* expression was significantly repressed in intestinal biopsy samples of CD patients in comparison to the healthy controls.^27^ Since *CRB3* is known to coordinate cell junction assembly and maintain the epithelial cell polarity, its reduced expression may lead to the delayed TJ formation and disruption of cell polarity, which is the most important characteristic of CD pathogenesis.^27^, ^28^ It is noteworthy that, *CRB3* duodenal mRNA expression demonstrated significant diagnostic value in differentiating our CD patients from the control group (*p* = .02). Further studies are required to validate the importance of this gene in this particular context.

There was not any significant difference in terms of *PRKCZ* expression between CD patients and controls in this study. Michaux et al. also in 2016, examined the location of essential apical polarity determinants, such as *Cdc42*, *Par6B*, *PKCζ/ι*, in microvillus inclusion disease patients like CD subjects and did not detect any changes in the location of polarity determinants in them.^29^ Schumann and colleagues conducted another study on healthy control subjects without any CD‐typical clinical and histological symptoms as well as CD patients with villous atrophy and a positive tissue‐transglutaminase‐ or endomysium‐IgA serology. They observed alterations in the expression level of *Par‐3* (another member of Par cell polarity family that form a cell polarity driving complex with atypical *PKC* and *Par‐6*) in the CD group and considered it to be the cause of paracellular leakage in active CD patients.^30^ Furthermore, studies involving cell culture and animal models of intestinal inflammation have suggested that pro‐inflammatory signaling downregulates the expression of atypical *PKC* (*aPKC*).^31^

The mRNA expression level of *LKB1* had a significant positive correlation with patients reported anemia and weight loss symptoms, which are known as most important CD‐related complications. According to the reports, *LKB1* through the mTOR signaling pathway plays an important role in the metabolism and production of red blood cells, which can justify the observed correlation.^32^ Moreover, *PRKCZ* and *LKB1* had significant negative correlations with the severity of intestinal tissue damage. Targeting these two genes to reach them to the control level can be considered as an important strategy for further studies with the aim of finding novel therapeutic strategies for controlling CD patients clinical and pathologic manifestations. There were significant correlations between *LKB1* and *SCRIB*, *LKB1* and *PRKCZ*, and *PRKCZ* and *SCRIB* duodenal mRNA expression, highlighting their importance in CD.

Moreover, we found the interactions between our selected genes and *MPP5* (*PALS1*), *STRADA*, *CAB39*, *VANGL2*, *FZD3*, *PRKAA2*, *F11R*, and *STK11IP*. *PALS1* is a member of the Crumbs polarity complex and has an important role in the establishment of cell polarity and biogenesis of TJs.^33^ *STRADA*, functioning as a pseudokinase, collaborates with *LKB1* to form a heterotrimeric complex, thereby activating *LKB1*. Additionally, *STRADA* plays a pivotal role in *LKB1*‐induced G1 cell cycle arrest.^34^ *CAB39* is known as a novel regulator of tumor metabolism in gastric cancer and can interact with *LKB1*‐*STRAD* complex leading to activation of *LKB1*.^35^ *STK11IP* may also regulate *LKB1* function by controlling its subcellular localization.^36^ *VANGL2* is one of the components of the planar cell polarity and is related to the tissue polarity pattern.^37^ *F11R* has an important role in the formation of epithelial TJ and paracellular permeability.^38^, ^39^ *FZD3* is highly correlated with colorectal cancer progression and its increased expression is reported in IBD colon tissue samples.^40^, ^41^ *PRKAA2* is linked to Peutz‐Jeghers syndrome, a condition characterized by the presence of hamartomatous polyps in the gastrointestinal tract.^42^

Current study has some limitations that should be acknowledged. First, the small sample size may limit the generalizability of the findings. Additionally, the recruitment of participants solely from a specialized research center may introduce selection bias. The study's focus on mRNA expression levels without functional analysis is another limitation. Consequently, the findings may not apply to other populations. Therefore, there is a need for larger, longitudinal studies involving diverse populations to gain a deeper understanding of the role of *SCRIB*, *PRKCZ*, *LKB1*, and *CRB3* in CD.

## 5 CONCLUSION

Intestinal epithelial apical–basal polarity is established by polarity complexes that interconnect with each other to maintain tissue homeostasis. However, until now little is known about the relative expression of polarity‐related genes in CD. Here, we provided expression patterns of the most important polarity genes in CD patients. Based on our analysis, *CRB3*, *LKB1*, and *SCRIB* were found to have significantly lower expression levels in CD patients compared to healthy controls, while *PRKCZ* showed no significant differences in expression between the two groups. *CRB3* duodenal expression had the potential to be introduced as a diagnostic biomarker for CD that larger cohort studies, including pediatric patients and using other biologic samples, are needed to achieve more insights in this regard. The study findings highlight the importance of apical–basal polarity and barrier integrity in the pathogenesis of CD, that may facilitate the planning of the future studies looking for finding innovative therapeutic strategies for CD. Understanding the molecular mechanisms underlying apical–basal polarity defects in CD could provide valuable insights for future therapeutic targets. Further research is warranted to validate and expand upon these findings, as well as investigate the potential interactions and pathways involving the identified genes.

## AUTHOR CONTRIBUTIONS

**Tannaz Taraz**: Data curation; formal analysis; investigation; methodology; project administration; software; visualization; writing—original draft. **Nastaran Asri**: Data curation; formal analysis; investigation; methodology; software; visualization; review and editing. **Ehsan Nazemalhosseini Mojarad**: Methodology; software; validation; investigation; data curation; review and editing. **Flora Forouzesh**: Project administration; data curation; review and editing. **Mostafa Rezaei‐Tavirani**: Formal analysis; software; review and editing. **Mohammad Rostami‐Nejad**: Data curation; supervision; validation; conceptualization; project administration; review and editing.

## CONFLICT OF INTEREST STATEMENT

The authors declare no conflict of interest.

## ETHICS STATEMENT

The study was approved by the Ethical Committee at the Research Institute for Gastroenterology and Liver Diseases, Shahid Beheshti University of Medical Sciences (IR.SBMU.RIGLD.REC.1401.002). Written informed consent was obtained from study subjects before enrollment.

## Supporting information

Supporting information.

## Data Availability

The data that support the findings of this study are available from the corresponding author upon reasonable request.
