# Supplementary material for: Intestinal mRNA expression analysis of polarity‐related genes identified the discriminatory ability of CRB3 as a diagnostic marker for celiac disease
Source: Immun Inflamm Dis. 2024 Feb 14;12(2):e1186. doi: 10.1002/iid3.1186 (PMC10865414; doi:10.1002/iid3.1186)
Supplement: Supplementary file 1 — Supporting information. [file IID3-12-e1186-s001.docx]

**Supplementary tables**

**Table S1.** Primers used in qPCR

| Primer  length | Primer sequence | Gene |
| --- | --- | --- |
| 20 | 5´GGACGACGAGGGCATATTCA3´ | SCRIB (F) |
| 20 | 5´CAGAGCCACACCATTCACCT3´ | SCRIB (R) |
| 20 | 5´TACATACATACCCCGTGCGT 3´ | CRB3 (F) |
| 19 | 5´AGAGGGCGGAGTCAAGAAG3´ | CRB3 (R) |
| 20 | 5´GCCGTGTTCATACTTGTCCG3´ | LKB1 (F) |
| 19 | TCAGACGCGAGTTCCAGTG 3´´5 | LKB1 (R) |
| 20 | 5´CTCCTGGTGCGGTTGAAGAA 3´ | PRKCZ (F) |
| 20 | 5´CTGGATGCCTGCTCAAACAC3´ | PRKCZ (R) |
| 21 | 5'- ATGTGGCCGAGGACTTTGATT -3' | B2M (F) |
| 21 | 5'- AGTGGGGTGGCTTTTAGGATG -3' | B2M (R) |

F: Forward; R: Reverse
